# GACAL: Conjecture-Based Verification (Competition Contribution)

**DOI:** 10.1007/978-3-030-45237-7_26

**Published:** 2020-03-13

**Authors:** Benjamin Quiring, Panagiotis Manolios

**Affiliations:** 8 Johannes Kepler University, Linz, Austria; 9 University of Birmingham, Birmingham, UK; Northeastern University, Boston, MA USA

## Abstract

GACAL verifies C programs by searching over the space of possible invariants, using traces of the input program to identify potential invariants. GACAL uses the ACL2s theorem prover to verify these potential invariants, using an interface provided by ACL2s for connecting with external tools. GACAL iteratively searches for and proves invariants of increasing complexity until the program is verified.

## 1 Verification Approach

GACAL is a tool for verifying reachability queries in C programs by iteratively and efficiently performing conjecture generation and conjecture verification. Conjecture generation involves searching through the space of possible conjectures using evaluation-based testing to identify likely-to-hold conjectures, and conjecture verification consists of using software verification technology to verify these conjectures. Our initial motivation was to develop a computational agent that can automatically complete the Invariant Game [1], in which players suggest invariants that are used by a reasoning engine to verify imperative programs, which we did with success- GACAL is a more fully developed form of the underlying conjecture generation ideas. This section presents a brief overview of GACAL's basic structure and methods for conjecture-based verification, and then discusses these, as well as associated challenges, in more depth. Section 2 provides information about the GACAL project, Section 3 provides an evaluation of GACAL, and Section 4 concludes this paper and discusses future work.

In GACAL, conjectures are potential invariants paired with program locations. Evaluation-based testing consists of evaluating possible invariants using execution-produced program traces. The ACL2s theorem prover [2] verifies conjectures using a graph representation of the input program. To search through the space of conjectures, GACAL first constructs a space of terms, which are C-expressions composed of the constants, variables, and arithmetic/bitwise operators in the program. Terms are combined using relational and logical operators to create possible invariants, and possible invariants which hold in all generated program traces are promoted to potential invariants and turned into conjectures. Discovered potential invariants are then analyzed using ACL2s and, if proven, used to verify the program. In the case that the program cannot be verified from the currently proven invariants, the above process is repeated: construct new, more complex, terms, find potential invariants via testing on program traces,

* Jury member: quiring.b@northeastern.edu prove potential invariants, attempt program verification, and repeat. At a high-level, this loop is the heart of GACAL's conjecture-based verification.

GACAL's approach to verification presents challenges which can be summarized into two categories: how to minimize the number of generated conjectures, and how to optimize the interactions with ACL2s. The techniques GACAL uses to address these challenges, as well as a more in-depth explanation of the previously mentioned methods are outlined below.

**Term and Invariant Construction** GACAL builds the space of terms by iteratively constructing all terms of a fixed size, where the size of a term is the number of constants, variables, and operators in that term. GACAL uses a collection of rewrite rules to filter the newly constructed terms: terms which can be rewritten to an equivalent form that has already been constructed are not kept. The size partial order on terms allows GACAL to perform rewriting effectively. Furthermore, the term constructor searches for new rewrite rules by evaluating and comparing terms under a set of random assignments to find pairs of equivalent terms. The discovered equivalences are generalized and turned into rewrite rules which are added to the collection of rewrite rules. We designed the rewriting techniques to have the property that all terms which cannot be rewritten are semantically distinct. In general, the term space is at least asymptotically exponential in size, and the rewriting techniques above, for the class of problems we consider, significantly improve the asymptotics.

Possible invariants are C-expressions of the form $x$ `==` $y$, $x$ `<` $y$, $x$ `<=` $y$, and $P$ `||` $Q$, where $x$, $y$ are terms and $P$, $Q$ are possible invariants. We allow multiple invariants to be associated with each program location, hence, we do not need explicit conjunction. We note that the space of possible invariants is closed under logical negation. GACAL filters out possible invariants which can be rewritten to an equivalent form that has already been created, reducing the size of the invariant space. The order the invariant space is searched over is deterministic and independent of the given program, and was chosen because it worked well for the benchmark programs. At a high level, GACAL inspects more specific invariants before more general invariants (e.g. $x$ `==` $y$ before $x$ `<=` $y$).

**Trace Generation** To produce traces through the program GACAL creates many initial program states which randomly seed the result of all nondeterministic behaviors that occur during execution of the program, making them deterministic. For example, a seeded pseudo-random number generator can obtain values for 'nondeterministic integer' expressions. The initial states are propagated through the program for a bounded number of steps, generating a set of states associated with each program location. These initial traces are not changed during the course of verification.

Testing on program traces is essential to GACAL's conjecture generation, but programs may, for example, contain loops with many iterations or not terminate, and so obtaining traces which correspond to complete program executions may be computationally infeasible or impossible. To address this, GACAL creates additional types of traces which approximate the input program's behavior. The first type of these traces generalizes large constants to small and/or nondeterministic values, which allows loops with originally many iterations to be completed. The second type uses the counter-example generation abilities of ACL2s [3,4,5] to generate states at any program location which satisfy all currently proven invariants at that location, which are then propagated through the program. As GACAL proves more invariants, it recomputes the second type of traces to obtain a better approximation of the program. Since invariants tested on these traces are later checked for correctness, the fact that the traces may not reflect the original program's behavior does not introduce unsoundness. The states from the above two methods are only used to test invariants at a program location if there are no states from the original traces produced for that location, and if traces cannot be found at all then GACAL assumes all invariants are potential.

**Conjecture Verification** To prove conjectures, GACAL uses an algorithm which takes previously proven invariants as well as currently unproven potential invariants and iteratively removes invariants which cannot be proven until it reaches a fixpoint. This process requires a large number of verification queries and for the majority of programs checking these queries using ACL2s is where the majority of execution time is spent. To improve the ability of ACL2s to reason about GACAL queries, we developed an arithmetic library consisting of ACL2s theorems about the GACAL-supported C operators. Additionally, GACAL caches previous queries and their results, which allows it to answer queries that are similar to cached queries, without using the theorem prover. Finally, GACAL saves counter-examples that ACL2s provides when it falsifies queries and uses them to falsify new queries.

## 2 Tool Setup and Software Project and Architecture

The competition submission[1] uses GACAL version 1.0. GACAL requires Python 3, Java, and Common Lisp, and the competition archive contains all files necessary to run GACAL without further installation. Other relevant information may be found in the README file. GACAL only competes in the C ReachSafety-Loops category. GACAL is maintained by Benjamin Quiring and Panagiotis Manolios, and is implemented primarily in Common Lisp. The external tools used by GACAL are the Eclipse CDT parser and the ACL2 Sedan [2]. GACAL is publicly available at https://gitlab.com/acl2s/conjecture-generation/gacal under a GNU GPLv3 license.

GACAL does not handle all C language features. Most importantly, GACAL does not handle arrays and types other than 32-bit unsigned and signed integers. There is no theoretical reason for this. GACAL does not correctly model C semantics for undefined behavior in signed arithmetic. There is a bug in the contest submission for translating goto statements into our graph representation of programs which affects a small number of benchmarks.

[1] Available at https://gitlab.com/sosy-lab/sv-comp/archives-2020 and Zenodo [6].

## 3 Evaluation

GACAL performs best on programs it can execute to completion because this allows us to produce high quality traces covering all program locations. When this is not the case, GACAL often creates false conjectures which lead to a large number of theorem prover queries. Additionally, we note GACAL's execution time depends on the size of the term and invariant spaces, which grow exponentially based on the number of program variables, constants, and operations. The current version of GACAL verifies 66 of the 109 benchmark programs it parses, and the top three tools on this distribution verified 102, 70, and 70. There was one program which no other tools could verify, though GACAL succeeded.

The core of GACAL consists of potential invariant generation using program traces and the rewriting methods as outlined above. We found that the addition of the arithmetic library is essential to our ability to reason about unsigned arithmetic and the mod operator, allowing GACAL to verify 10% more total programs (which deal primarily with the listed features) and cuts the average time to query ACL2s by 33% on the verification queries which were not caught by the caching. We found that the additional trace generation methods did not significantly increase the number of programs that were verified, though they did decrease the average time for verifying a program. The caching of proof results and counter-examples is able to eliminate 85% of all verification queries from being submitted to ACL2s for checking, which increases the number of programs which are verified by over 10% and almost halves the average cost to verify a program. The caching methods also amplifies the benefits of the library and extra trace generation methods.

## 4 Conclusions and Future Work

There are many ways to improve GACAL, including incorporating classical analyses such as range analysis, abstract interpretation, symbolic evaluation, etc, as well as handling a larger subset of the C language. Another improvement to GACAL is to perform the search for disjunctive invariants more efficiently; currently GACAL often finds many potential but false disjunctive conjectures, which result in a large number of verification queries. One way to improve the search may be to analyze the program to find meaningful hypotheses, which could considerably lower the number of tested and generated conjectures.

We believe that GACAL provides evidence that our conjecture-based verification techniques can be used to improve current software verification tools, as we were able to verify a competitive number of programs on the distribution we parse and we were able to verify a program that all other tools failed to verify, despite not using any of the classical analyses identified above.
